# Safety Priorities and Underestimations in Recreational Scuba Diving Operations: A European Study Supporting the Implementation of New Risk Management Programmes

**DOI:** 10.3389/fpsyg.2018.00383

**Published:** 2018-03-23

**Authors:** Serena Lucrezi, Salih Murat Egi, Massimo Pieri, Francois Burman, Tamer Ozyigit, Danilo Cialoni, Guy Thomas, Alessandro Marroni, Melville Saayman

**Affiliations:** ^1^Tourism Research in Economics, Environs and Society, North-West University, Potchefstroom, South Africa; ^2^DAN Europe Research Division, DAN Europe Foundation, Roseto degli Abruzzi, Italy; ^3^Department of Computer Engineering, Galatasaray University, Istanbul, Turkey; ^4^DAN Southern Africa, Midrand, South Africa; ^5^DAN USA, Durham, NC, United States

**Keywords:** buddy system, accessories, incident, accident, awareness, prevention campaign, training, dive centre

## Abstract

**Introduction:** Scuba diving is an important marine tourism sector, but requires proper safety standards to reduce the risks and increase accessibility to its market. To achieve safety goals, safety awareness and positive safety attitudes in recreational scuba diving operations are essential. However, there is no published research exclusively focusing on scuba divers’ and dive centres’ perceptions toward safety. This study assessed safety perceptions in recreational scuba diving operations, with the aim to inform and enhance safety and risk management programmes within the scuba diving tourism industry.

**Materials and Methods:** Two structured questionnaire surveys were prepared by the organisation Divers Alert Network and administered online to scuba diving operators in Italy and scuba divers in Europe, using a mixture of convenience and snowball sampling. Questions in the survey included experience and safety offered at the dive centre; the buddy system; equipment and accessories for safe diving activities; safety issues in the certification of new scuba divers; incidents/accidents; and attitudes toward safety.

**Results:** 91 scuba diving centres and 3,766 scuba divers participated in the study. Scuba divers gave importance to safety and the responsiveness of service providers, here represented by the dive centres. However, they underestimated the importance of a personal emergency action/assistance plan and, partly, of the buddy system alongside other safety procedures. Scuba divers agreed that some risks, such as those associated with running out of gas, deserve attention. Dive centres gave importance to aspects such as training and emergency action/assistance plans. However, they were limitedly involved in safety campaigning. Dive centres’ perceptions of safety in part aligned with those of scuba divers, with some exceptions.

**Conclusion:** Greater responsibility is required in raising awareness and educating scuba divers, through participation in prevention campaigns and training. The study supports the introduction of programmes aiming to create a culture of safety among dive centres and scuba divers. Two examples, which are described in this paper, include the Hazard Identification and Risk Assessment protocol for dive centres and scuba divers, and the Diving Safety Officer programme to create awareness, improve risk management, and mitigate health and safety risks.

## Introduction

### Problem

Scuba diving is a sport and recreational activity that has become one of the most important marine tourism sectors globally, with up to 1,000,000 certifications issued annually (Garrod and Gössling, 2008; Musa and Dimmock, 2013; Dimmock and Musa, 2015; PADI, 2017). Over time, training agencies have developed relatively safe standards for a subgroup of leisure divers who are not risk seekers, but rather passive observers of the natural environment and marine life (Whatmough et al., 2011). The development of safe standards has gone hand in hand with the mass commercialisation of the scuba diving sport (Dimmock and Cummins, 2013). In turn, the scuba diving industry has evolved to include a support system around people practising the sport (MacCarthy et al., 2006). Certifying agencies have created new education and training packages, and travel agencies and destinations have been accommodating the necessities of a more diversified market (Dimmock et al., 2013). Safety has become paramount to ensure that scuba diving could be increasingly accessible to new markets, new destinations could become within reach of scuba diving with minimal risks, and scuba diving could translate into a safe and relaxed adventure, in line with increasing market demands (Dimmock and Musa, 2015). Safety organisations, including the Divers Alert Network (DAN), are dedicated to research on scuba diving safety and medicine, campaigning, emergency medical assistance, education, prevention, mitigation, accessories, and insurance for diving operations and scuba divers.

Today, scuba diving is a relatively safe sport (Wilks and Davis, 2000; Divers Alert Network [DAN], 2017), meaning that there is still a residual risk, which is considered a so-called ‘accepted residual risk.’ So, accidents do still happen in scuba diving. In addition, safety issues related to scuba diving still receive negative attention by the media globally (Marroni et al., 1994; Wilks and Davis, 2000). In 2017 alone, there were at least 30 news headlines on scuba diving fatalities, featuring in websites such as bbc.com, cbc.ca, foxcarolina.com, and independentmail.com. These issues, coupled with the increased accessibility of the sport in new markets such as holiday makers, disabled people and children, calls for constant attention to and improvements in safety standards (Dimmock and Musa, 2015).

**FIGURE 1:**
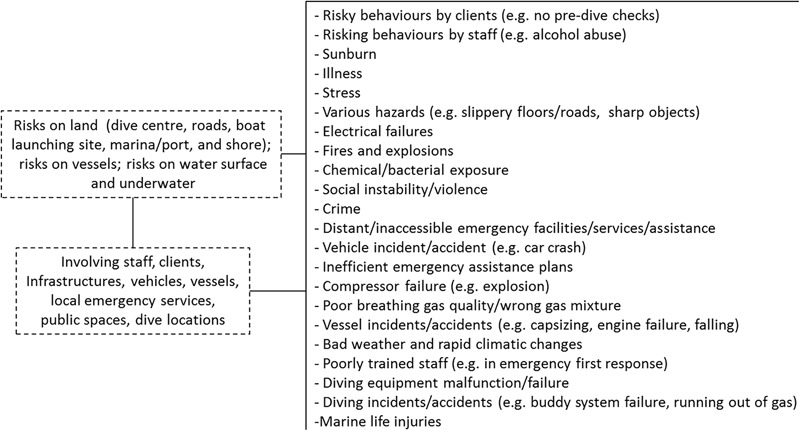
Risks associated with recreational scuba diving operations. Source: Authors.

There is also an important difference to consider between professional and recreational diving. On the one hand, professional diving has established risk assessment, mitigation programmes, codes of practise and regulations since long ago and as a consequence, it has become a safe profession. On the other hand, recreational diving is based on self-responsibility, and therefore risk awareness and attitudes are determining factors for triggering dive accidents. Recreational scuba diving operations are exposed to a series of risks (**Figure 1**). These can develop into liability, incidents/accidents, injury or death of people affected. Risks must be considered for various locations and can involve a number of people, infrastructure, vessels, vehicles, the environment and services. Although certifying and safety agencies are in possession of data regarding risks that are reported directly by the recreational scuba diving industry, there is no published research focusing exclusively on scuba divers’ and dive centres’ perceptions toward safety. As there is still evidence of avoidable accidents in recreational diving in spite of a well-established education by the training agencies, we believe that this training is mainly focussed on skills and appropriate materials, and that more awareness of and proper attitude toward safety would help to reduce the number of such incidents and accidents.

### Aims and Objectives of the Study

This is the first study that assesses safety perceptions in recreational scuba diving operations. The aim was to collect data on the safety perceptions of dive centres and scuba divers, to guide decision making concerning safety in scuba diving operations. Bottom–up information, generated not only by dive centres and professionals, but also by scuba divers, is important to ensure that perceptions of various role players in the recreational scuba diving industry are aligned, and to support and enhance the implementation of safety management programmes in recreational scuba diving operations.

More specifically, information was collected from dive centres in Italy and scuba divers in Europe for four purposes: to identify general safety perceptions at dive centres and among scuba divers; to assess whether information from dive operators aligns with information from scuba divers and address gaps; to propose ways to educate scuba divers on the real risks of diving; and to verify if the results from the study would justify introduction of programmes of risk mitigation and risk prevention in recreational scuba diving.

### Safety Attitudes and Behaviour in Recreational Scuba Diving Operations

The literature offers elements supporting this investigation. On the one hand, these elements shed a positive light on the relationships between scuba diver training and safety behaviour, the link existing between environmental protection and safety, and the general safety attitudes and behaviour of scuba divers. For example, recent work by Ong and Musa (2012) shows that scuba divers see themselves as very responsible under water, checking their underwater position/orientation, carrying a surface marker buoy as a safety accessory, and monitoring their diving depth at all times. In addition, scuba divers with better knowledge of diving practises and diving skills (e.g., buoyancy) show safer behaviour under water. Finally, scuba divers who are more environmentally responsible (avoiding contact with the substrate and with marine life) are safer as well. A study by Merchant (2011) relays the attitudes of scuba divers toward the use of equipment for safety; scuba divers appreciate the protective role of wetsuits, boots, gloves and hoods against hazardous interactions with marine life and cold temperatures. A recent investigation by DAN Europe, on the diving profiles of 39,000 dives from over 2,600 scuba divers in Europe, shows that scuba divers dive within the recommended ‘safe zone’ (not exceeding 30 m in depth) and that their average ascent rate is lower that the recommended safe speed; thus, scuba divers dive conservatively (Cialoni et al., 2017). These findings suggest that scuba divers tend to follow recommendations made by known diving safety agencies, as well as a tendency toward best practise and safety prioritisation.

Generally, divers’ positive attitudes toward safety are linked with the performance and views shared by service providers, including dive centres. Research by O’Neill et al. (2000) and Ince and Bowen (2011) demonstrates how the attention to safety paid by dive centres has a strong impact on clients’ perceptions, increasing satisfaction and conveying a feeling of trust. The studies show that, when selecting a dive centre, scuba divers give priority to its safety record and approach; in addition, they regard the appearance of the dive centre’s premises, vehicles, staff and equipment as important in terms of assurance and safety standards. Andy et al. (2014) highlighted that, according to scuba divers, diving skills, including those in managing problems and those in leading dives, are crucial competencies that dive guides need to possess.

On the other hand, scuba divers may engage in risky behaviour, show limited interest in safety procedures and accessories, and are negligent in reporting accidents and incidents. The British Sub-Aqua Club has been stating for decades that generally, most scuba diving accidents would be easily avoidable if simple diving practise principles were followed (Cumming and Watson, 2017). Dive centres may be at fault by not enforcing safety regulations, not pursuing the best approach in managing risks, and sharing skewed views on safety with clients. A study by Bonnet et al. (2008) reports that 50% of divers interviewed in France regularly took risks while scuba diving, diving below depths allowed by their respective scuba diving certifications, and performing several dives in succession, resulting in nitrogen saturation. Recent work by Lafère et al. (2017) reports on how scuba divers can continue to adopt risky diving behaviours even after experiencing decompression illness, and after being diagnosed with risky physiological conditions and advised with contra-indications. The study highlights the importance of including the scuba diver psychological profile in diving risk research. Research by Wilks and Davis (2000) also argues that there are issues with the reporting of fatal and non-fatal accidents and incidents in the recreational scuba diving industry, due to limited access to information held by hospitals and medical surgeries. Of the reported accidents, some are not properly identified from hospital records, with the exception of decompression illness. Other literature highlights the controversial views by scuba divers with regard to the buddy system, which is normally accepted as a standard safety procedure of monitoring one another during diving, yet is either dismissed or disregarded by a good proportion of scuba divers (Coleman, 2007). The failure of the buddy system is reported as one of the main causes of accidents in scuba diving, as well as featuring consistently in scuba diving fatalities (Ranapurwala et al., 2017).

## Materials and Methods

### Research Introduction and Handling of the Participants’ Data

The research followed a quantitative, descriptive and non-experimental method of data collection, deploying a structured questionnaire survey for scuba diving operators and scuba divers. However, the first phase of the research was characterised by a qualitative pilot study collecting preliminary data to guide the structuring of the survey. With regard to the handling of the participants’ data at any phase during this study, there was no instance in which the identity of the participants had to be made known; therefore, all data were treated anonymously. Further, no sensitive data were requested throughout the research, respecting the privacy of the participants. Last, by agreeing to take part in the research, the objectives of which were always clearly outlined upon invitation, the participants provided an informed consent and were free to leave the research, either by interrupting an ongoing interview or by deciding not to complete a questionnaire survey, at any moment.

### Pilot Study for Survey Design

The pilot study took place in the Portofino Marine Protected Area (MPA), which is located in north-western Italy in the Liguria Sea. This location was selected based on its long scuba diving history and its popularity as a recreational scuba diving destination in Europe (Lucrezi et al., 2017). The MPA counts approximately 20 scuba diving centres distributed among various towns, and up to 80,000 dives are logged in the MPA annually (Pedemonte, 2017). During November 2015, dive centres in the Portofino MPA were visited by staff of DAN, and invitations to operators and scuba divers to participate in a face-to-face interview were extended. Three dive centres and 17 scuba divers participated in the interviews, which covered aspects including demographic profile; scuba diving experience; safety attitudes; scuba diving incidents/accidents; and risk prevention. Interview responses were transcribed verbatim and subjected to thematic analysis to guide the structuring of the questionnaire survey for the second phase of the research.

### Survey Design and Structure

Based on the data collected in the first phase of the research, two structured questionnaire surveys were designed to target both scuba diving centres in Italy and scuba divers in Europe. Italian dive centres were selected for this study for two main reasons: Italy counts the greatest number of dive centres with active DAN membership in Europe; and Italy represents the main case study of the Green Bubbles project^1^, a research project on sustainable scuba diving funded by the European Commission. The survey was prepared by medical and engineering staff at DAN (Europe and Southern Africa) in collaboration with engineers, marine biologists and environmental scientists participating in Green Bubbles.

The surveys included mostly close-ended questions, deploying a five-point Likert scale of importance (1 = No importance to 5 = Very important) or multiple-choice questions. The questionnaire directed at dive centres included questions on: dive centre characteristics; the importance of experience and safety aspects offered at the dive centre; influencing factors in the formation of dive groups and buddy pairs at the dive centre; the importance of equipment and accessories for diving; the importance of various aspects in the certification of new scuba divers; incidents/accidents at the dive centre; and safety attitudes. The questionnaire directed at scuba divers included questions on: demographic details and scuba diving experience; the importance of safety aspects when choosing a dive centre; influencing factors when choosing buddies; the importance of various forms of safety behaviour as a scuba diver; the importance of equipment and accessories for diving; scuba diving incidents/accidents; and safety attitudes.

### Second Pilot Study and Final Administration to Dive Centres and Scuba Divers

Questions in both surveys were checked by a hired statistician for statistical validity. Following this cheque, the survey directed at scuba divers was tested through a pilot study, in which data were collected using a mixture of convenience and snowball sampling techniques. In the pilot study, the survey was made available online through KwikSurvey, and promoted for 10 days in Italy and Turkey using direct emails sent to scuba divers registered with DAN Europe, the DAN Europe web site, the social networks (Facebook), and newsgroups. Following the pilot study, both surveys were finally launched. The population of dive centres in Italy is approximately 1,000 (Italia Sub, 2018; C. Pellegrini personal communication), and that of certified scuba divers in Europe is 3,000,000 (European Underwater Federation, 2018), although it is not known how many of these individuals are active divers. Therefore, a mixture of convenience and snowball sampling (inviting centres and divers through the DAN Europe membership network and social networks like Facebook and Twitter) was adopted to maximise the outreach of the surveys to these populations. The surveys were made available online through KwikSurvey and Survey Monkey, in Italian for the dive centres, and in Italian, English, French, German, Spanish and Turkish for the scuba divers in Europe. Both surveys were launched at the end of 2015 and closed at the end of 2016.

### Data Analysis

Data were captured in Microsoft Excel (2010) and all analyses were performed with Statsoft Statistica software (Version 13.2, 2016). Graphs were created with GraphPad Prism (Version 5, 2007). The profile of the participants (dive centres and scuba divers) and their responses were outlined through descriptive statistics, breakdown statistics and frequency tables. Responses from both groups (dive centres and scuba divers) were represented separately to identify commonalities and divergences. The results were worked up in order to be used later for actions concerning safety at dive centres and among scuba divers.

## Results

### Pilot Study for Survey Design and Second Pilot Study

The majority of the participants in the pilot study (70%) were male and recreational scuba divers (95%), with some also possessing technical qualifications. About 80% had logged 51–200 total dives, and an additional 15% had logged over 200 dives. From the thematic analysis of the interviews with the participants (pooling views from dive centre operators and scuba divers), it is evident that the participants tended to be ‘safe’ divers and/or to promote ‘safe’ diving: they dived at depths ranging between 21 and 40 m; they dived mostly with a buddy; they towed a dive flag and used a surface marker buoy when needed; and they also carried an underwater torch with them. However, the participants also made a number of statements which highlighted the need for better investigation of perceived risks and safety procedures, risk identification and mitigation, and emergency action/assistance plans. For instance, although the majority of the participants claimed never to have experienced an incident/accident while scuba diving, a good proportion (30%) had mentioned at least one type of incident/accident. When asked what their greatest fears were in relation to scuba diving activities, the participants provided answers including drowning, decompression illness, and boating accidents. Last, when asked whether they were aware of any formal way of assessing risks and identifying hazards, the participants answered negatively. These answers provided the foundation for the development of the survey which was tested through the second pilot study.

A total of 204 scuba divers from Turkey and 187 scuba divers from Turkey participated in the second pilot study. Since the results from the second pilot study are similar to those of the final survey directed at scuba divers, the results from the second pilot study are not reported.

### Final Survey to Dive Centres and Scuba Divers

A total of 91 scuba diving centres from Italy and 3,766 scuba divers from Italy, Germany, France and Turkey, among other countries, participated in the questionnaire surveys. The proportion of dive centres participating in the survey represented a suitable sample with a confidence level of 95% and a confidence interval of 9.8. The proportion of scuba divers participating was a suitable sample with a confidence level of 99% and a confidence interval of 2.1.

All dive centres catered for recreational divers, although 60% also accommodated technical and free divers. A similar proportion accommodated scuba divers with disabilities. Most dive centres offered boat dives (over 60%) and shore dives (33%). The scuba divers were mostly male (75%) and middle aged (57% were 41–60 years old) with a good level of experience. Nearly half of them had logged 100–500 dives, and their average certification level was advanced (43%). Good proportions were also instructors (31%). Whereas the majority (over 70%) of the divers were recreational, 34% possessed a technical qualification, and 23% defined themselves as ‘technical divers.’ Nearly all had dived outside their country of origin. The majority (58%) preferred to dive at depths between 19 and 30 m, with an additional 20% preferring to dive down to 40 m. Only 10% preferred depths beyond 40 m. Most dives were from a boat (57%).

**FIGURE 2:**
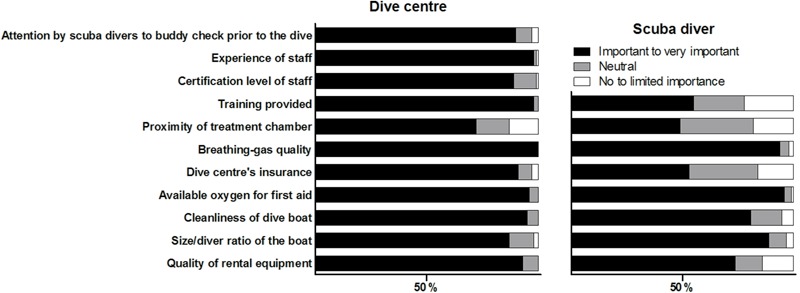
The importance of experience and safety aspects that are offered at the dive centre according to the dive centres **(left)** and according to scuba divers **(right)**.

A summary of important experience and safety aspects that should be provided at the dive centre, according to the two groups (dive centres and scuba divers), is represented in **Figure 2**. The most important aspect to all dive centres was breathing-gas quality, followed by staff experience, training provided, and available oxygen for first aid. Although the proximity of a treatment chamber was relevant for over half of the dive centres, it received the lowest importance score. In line with dive centres, scuba divers believed that breathing-gas quality and available oxygen for first aid were the most important elements to consider when choosing a dive centre. They also gave the least importance to the proximity of a treatment chamber, and less importance to training provided and whether the dive centre had insurance.

**FIGURE 3:**
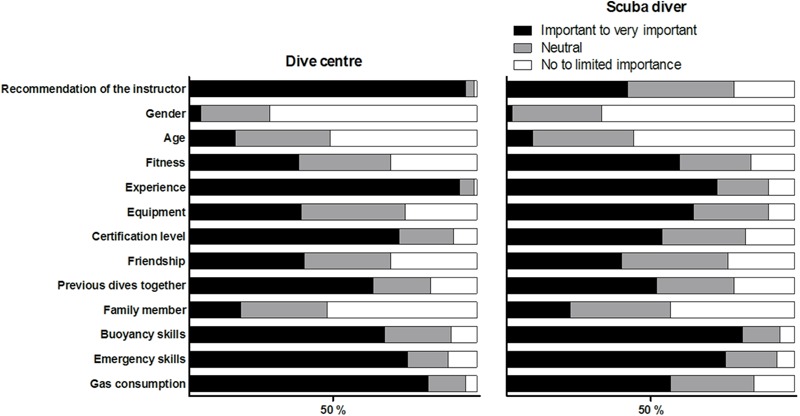
Influencing factors in the formation of dive groups and buddy pairs at the dive centre according to the dive centres **(left)** and according to scuba divers **(right)**.

A summary of influencing factors in the formation of groups and buddy pairs according to dive centres and scuba divers is provided in **Figure 3**. Both groups agreed that gender, age, family relations and friendship were not influential. Dive centres believed recommendations by the instructors, experience and gas consumption of a potential buddy to be the best elements to consider. Scuba divers gave buoyancy skills, emergency skills and experience of a potential buddy the greatest importance, and did not think of instructors’ recommendations as influential.

**FIGURE 4:**
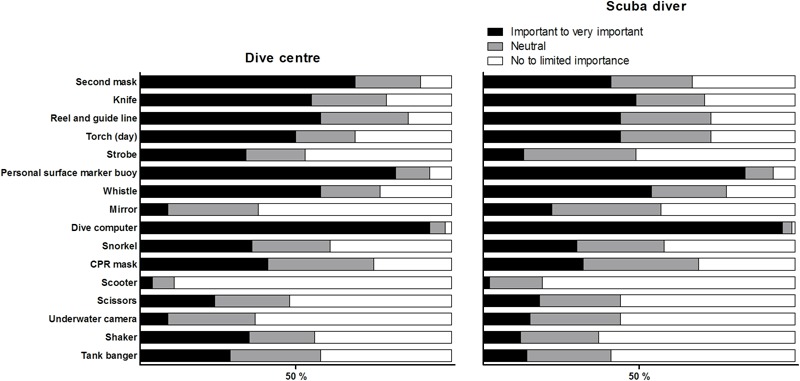
The importance of various types of equipment and accessories for diving activities according to the dive centres **(left)** and according to scuba divers **(right)**.

A summary of important accessories for diving according to dive centres and scuba divers is provided in **Figure 4**. Both groups considered the dive computer and the personal surface marker buoy as the most important accessories. Whereas over 50% of the dive centres gave importance to other accessories also, including a backup mask, whistle, knife, and a reel and guide line, scuba divers gave less importance to other accessories, with the exception of a whistle.

**FIGURE 5:**
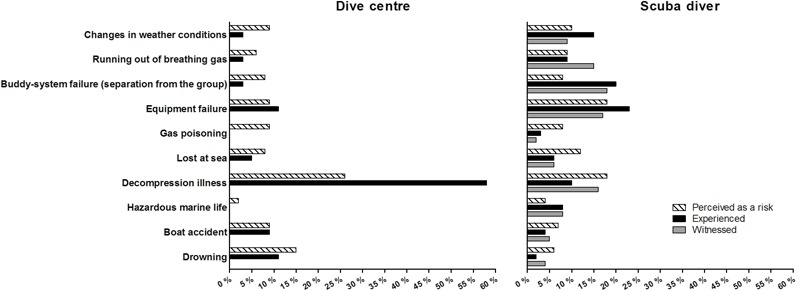
Incidents or accidents related to scuba diving activities perceived as a risk and reported by the dive centres **(left)** and perceived as a risk, experienced and witnessed by scuba divers **(right)**.

About 30% of the dive centres had dealt with scuba diving incidents/accidents, mostly with regard to decompression illness (60%), but also involving drowning (11%), equipment failure (11%), and boat accidents (9%; **Figure 5**). Dive centres perceived these events as the greatest risks in scuba diving (**Figure 5**). About 30% of the scuba divers had experienced a diving incident/accident, and twice this proportion had witnessed a diving incident/accident. Experienced and witnessed incidents/accidents involved equipment failure and splitting of the buddy pair (**Figure 5**). Other relevant incidents/accidents involved changes in weather conditions and interactions with hazardous marine life (**Figure 5**). Scuba divers were in greatest fear of equipment failure and decompression illness, but they were least concerned about hazardous marine life and the risk of drowning (**Figure 5**).

**FIGURE 6:**
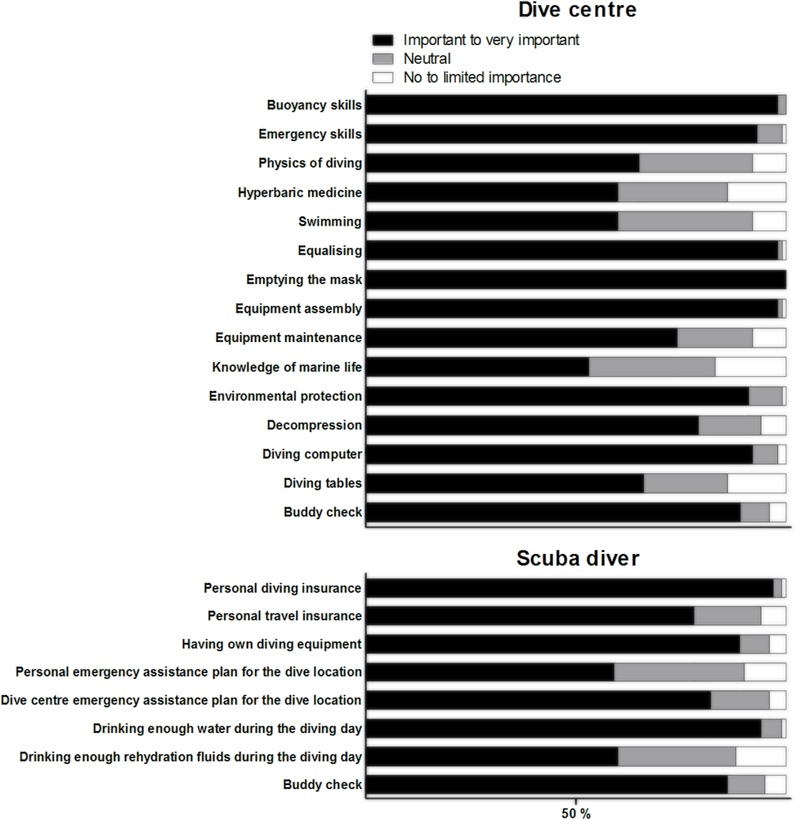
The importance of various aspects in the formation of new scuba divers according to the dive centres **(top)** and the importance of various forms of safety behaviour according to scuba divers **(bottom)**.

A summary of important aspects in the certification of new scuba divers, according to the dive centres, is provided in **Figure 6**. Most dive centres gave great importance to the basic aspects of diver training, particularly exercises such as clearing the mask, equalising, equipment assembly and buoyancy skills. They also valued emergency skills, learning how to use the dive computer, the buddy cheque and the importance of environmental protection. Although all dive centres had emergency action/assistance plans in place, about 10% did not have an active insurance plan for diving accidents and professional liability. They were actively involved in some safety campaigns, mostly related to the prevention of propeller incidents (33%), breathing-gas quality (32%) and keeping hydrated throughout the diving day (24%).

**Figure 6** also provides information on the attitude toward safety by scuba divers. Most scuba divers felt that being in possession of dive insurance and drinking enough water during the diving day are very important safety aspects of being a diver. They also gave importance to possessing one’s own diving equipment and to the buddy cheque. Nearly all dived with their personal equipment. All but 15% dived with a buddy; when doing a buddy cheque, 44% performed six or more controls, although a high proportion (55%) made fewer cheques and 1% made none. Divers gave importance to relying on the dive centre’s emergency action/assistance plans for a dive location. Although over 80% possessed active diving insurance, a smaller percentage (60%) had a personal emergency action/assistance plan in place.

## Discussion

### Safety Perceptions: Recommended Actions

Below is a discussion of the results of this study, accompanied by a description of relevant actions to be taken. These actions, as well as the results they are based on, are listed in **Table 1**. The scuba divers were mostly middle-aged men with considerable diving experience and thus likely to engage in intensive, although not necessarily technical, diving activities. These divers valued the ability to rely on the dive centre’s emergency action/assistance plans for a dive location. Given that ageing increases medical risks associated with scuba diving activities (Smerz, 2006; Ardestani et al., 2015), scuba diving operations face important implications for safety and risk management. The dive centres participating in this study offer a variety of services and cater for divers of different backgrounds and experience. However, the demographic profile of the participating scuba divers, and age range in particular, calls for a comprehensive understanding of risks associated with ageing in scuba diving, prevention actions and campaigns, and *ad hoc* emergency action/assistance plans, both on vessels and on land. Organisations such as DAN have been promoting publicly accessible campaigns of prevention and information online for ageing divers (Divers Alert Network [DAN] Europe, 2017c). However, more focus must be given to the matter, particularly considering that scuba divers of older generations may not be searching for safety information on the web but rather look for alternative, more accessible sources of information, such as the dive centre or magazines.

**Table 1:** Summary of actions based on the results from questionnaire surveys with dive centres and scuba divers.

Main results	Recommended action
Main market being characterised by middle aged people	Ageing and scuba diving information and prevention campaigns; *ad hoc* plans in place by dive centres
Main market being characterised by males	Understand lack of female participation in scuba diving and provide solutions
Limited participation by youth in scuba diving	Scuba diving education in schools
Limited participation of beginners in Citizen Science and research; no communication channel established with beginners	Introduce Citizen Science as part of ‘end of course’ package for beginners
Importance given by scuba divers and dive centres to breathing gas quality and to oxygen availability for first aid	Obtain support of divers for air quality control and on-board oxygen safety campaigns; ensure dive centres understand benefits of air quality analysis; ensure dive centres understand the importance of having oxygen on board
Personal emergency action/assistance plan underestimated by scuba divers	Develop a web based tool or mobile app providing a step by step guide for divers to have their own emergency action/assistance plan
Importance given by scuba divers to buoyancy skills and gas consumption when choosing as buddy; importance given by diving centre to buoyancy skills training	Promote buoyancy skills training and events
Importance given by scuba divers and dive centres to dive computer and surface marker buoy	Add a section on generic dive computer use together with classical decompression management chapters of training packages; more attention toward surface marker buoy deployment during training dives
Value of some safety-related accessories being underestimated by scuba divers	Add a dedicated section on safety-related accessories in equipment chapters of training packages
Value of daily fluid intake for rehydration being underestimated by scuba divers and dive centres	Hydration campaigns
Limited participation by dive centres in propeller injury and boat accident prevention campaigns	Propeller injury campaigns
Limited attention given by scuba divers and dive centres to hazardous marine life risks	Promotion of training courses for hazardous marine life injuries
Decompression illness perceived as top risk by scuba divers and dive centres and experienced as top accident/incident by dive centres	Safety campaign about decompression illness
Reported experienced and witnessed accidents by scuba divers greater than accident reports by scientific organisations	Encourage and facilitate dive incident and accident reporting
Equipment failure both experienced and perceived as a risk by scuba divers; importance given by scuba divers to equipment when choosing a buddy	Development of preventive maintenance programmes
Running out of gas and buddy separation either experienced or witnessed as top accidents/incidents; drowning, however, not perceived as a risk	Development of innovative devices to prevent/alert running out of gas, drowning, and buddy separation
Being lost at sea perceived as top risk by scuba divers	Development of innovative tools to prevent cases of lost divers at sea
Importance given by scuba divers and dive centres to pre-dive buddy cheque, but buddy cheque controls underestimated by scuba divers; buddy system failure experienced and witnessed as top accident/incident by scuba divers	Establish standard pre-dive buddy cheque procedures with the consensus of major dive organisations

Data on the profile of the scuba divers confirmed the limited participation of female persons and youth in the sport, which is a common finding in scuba diving research (Garrod, 2008). The lack of participation by younger people in scuba diving activities, which is reflected in recent statistics from international certifying agencies like PADI (2017), can be addressed through scuba diving education campaigns in schools, an initiative that is gaining momentum in Italy (Progetto Scuola D’Amare, 2017). The re-inclusion of female persons in the scuba diving market can be foreseen as difficult, due to the withdrawal of women from the market following maternity. However, marketing strategies are being tested to ease such re-inclusion through the participation of children in educational activities at the dive centre while parents enjoy scuba diving (C. Cerrano, personal communication). The studied population reflected the actual population of members in DAN Europe (Cialoni et al., 2017; M. Pieri, personal communication), which was the main vector of promotion of the questionnaire survey. However, the limited participation by beginners in the study was evident. This limitation may be a result of improper communication between the organisation and beginners. Providing an understanding of the importance of participating in scientific research to improve safety standards in scuba diving should be part of the ‘end of course’ package for beginners. This could motivate young divers to participate in Citizen Science to support safety policy.

Dive centres and scuba divers acknowledged breathing-gas quality and available oxygen for first aid as the top safety services to be provided by dive centres, in line with other research (O’Neill et al., 2000; Ince and Bowen, 2011). Consequently, receiving the support of dive centres and scuba divers for air quality control and on-board oxygen safety campaigns becomes a priority for safety agencies (Divers Alert Network [DAN] Europe, 2017c). A low proportion of dive centres seems to be actively involved in safety campaigns focusing on breathing-gas quality, whereas safety campaigns on oxygen on board vessels were not mentioned by the dive centres in this study. Dive centres need to be educated on the benefits of safety campaigning (e.g., on the risks of dehydration and propeller injuries). Safety campaigns covering different topics can either be a free initiative of the dive centre as well as an event promoted by safety organisations with the support of dive centres. Examples of the benefits of safety campaigning include better awareness and safety behaviour among staff and clients, and increased satisfaction and loyalty of clients.

Dive centres and scuba divers gave little importance to the proximity of a treatment chamber. In other contexts, such as in the Caribbean and the United States, scuba divers often wish to know where the nearest chamber is (F. Burman, personal communication). The perceptions of participants in this study could be a result of hyperbaric treatment being a worst case scenario that is out of dive centres’ control, in comparison with other scenarios involving divers and dive centres more directly. They could also be due to the requirement for recompression being included in emergency action/assistance plans. Scuba divers also gave limited importance to whether a dive centre had valid insurance for diving accidents; they felt that the ability of the dive centre to offer emergency action/assistance would be more useful. Although the two elements appear to be disconnected, emergency action/assistance plans offered to divers by dive centres may be enhanced by insurance policies. It is important to raise awareness on the connexion between a dive centre’s insurance and efficient emergency action/assistance plans. Paradoxically, scuba divers possessed active dive insurance, although they did not have a personal emergency action/assistance plan in place. This result may suggest that scuba divers do not take personal responsibility for safety and emergency action/assistance, and rather lay it on the dive centre and on safety agencies. Based on these outcomes, it is important to educate divers on assuming more responsibility in planning their own emergency response. The development of a web-based tool or mobile application, providing a step-by-step guide for divers to follow their own emergency action/assistance plan, seems like a reasonable approach for the diving community, which is becoming increasingly technology-driven.

Although dive centres and scuba divers did not agree on what is influential in the formation of buddy pairs and groups, all elements considered are worthy of attention. Experience of a potential buddy was given great importance by both groups. Scuba divers gave also importance to the buoyancy skills and emergency skills of a potential buddy (see also Andy et al., 2014). Even though dive centres felt that gas consumption would be more influential compared with these elements, they gave great importance to the latter as part of diver training. These results suggest how diver training should be restructured to give more attention to these aspects (Hammerton, 2017). When this is not possible, training should be integrated with events and workshops organised by safety and training agencies to raise awareness regarding buoyancy control and basic emergency first response. Examples include the Master Trim competition and the Oxygen Provider course launched by DAN Europe (Divers Alert Network [DAN] Europe, 2017a,b).

Modifications to diver training are also recommended in light of results related to diving accessories. Scuba divers and dive centres agreed that the dive computer and surface marker buoy are the most important accessories for scuba diving activities (Ong and Musa, 2012). This emphasis should be supported by re-categorising these accessories as ‘must have’ equipment, and by providing more information and training on their use. This can happen through the addition of a section on dive computer use together with classical decompression management chapters in training packages, and by dedicating more time to surface marker buoy deployment during training dives. Although dive centres appreciated the utility and value of other accessories, scuba divers did not. A dedicated section on safety accessories should be considered as a valuable addition to equipment-related chapters in scuba diver training packages.

The results of this study highlight limited participation in safety campaigns, including hydration campaigns, prevention of propeller incidents, and breathing-gas quality control. This was reflected in the little value given to aspects like daily fluid intake for rehydration during the diving day, perceptions of decompression illness, and limited attention given to marine life injuries. The overarching actions that can be taken are the promotion of safety campaigns and obtaining the support of dive centres to raise awareness (Divers Alert Network [DAN] Europe, 2017c). Campaigns on decompression illness should place an emphasis on statistics to reassure divers, yet retain key messages on the potential risks of the disease among scuba divers (Cialoni et al., 2017). The limited attention given to hazardous marine life can be addressed by information and the promotion of training courses for handling hazardous marine life injuries.

The data collected on perceived risks, incidents and accidents highlight the importance of safety campaigns to answer to the concerns of the diving community as opposed to top–down approaches that impose safety rules. Top–down approaches and regulations can make some sectors of the scuba diving industry (e.g., professional diving) very efficient. However, this system can only work if there is a legal obligation to follow such regulations. Recreational diving has no such regulations at all in many countries, and therefore the only alternative to achieve the same goal (acceptable risk and safe practise) is education and campaigns for awareness of risks, mitigation and proper attitudes toward safety. The data also point to communication and reporting gaps between the diving community and safety agencies. Figures of reported incidents/accidents that are either experienced or witnessed by scuba divers, as well as those experienced by dive centres, are noteworthy and greater than those featuring in accident reports by scientific organisations (Wilks and Davis, 2000; Cialoni et al., 2017). This gap needs to be addressed by encouraging and facilitating the reporting of dive incidents and accidents to safety agencies.

Scuba divers experienced equipment failure more frequently and perceived it as a greater risk compared with other incident/accident types; this result, reflected in the prime importance of equipment when selecting a buddy, is in line with other findings (O’Neill et al., 2000; Ince and Bowen, 2011). It is critical to give scuba divers better control and understanding of their equipment, together with the assurance that possible incidents/accidents would be faced with minimal safety repercussions. Preventive maintenance programmes run by safety organisations and certifying agencies and supported by scuba diving centres could serve these purposes. The programmes could be divided into levels, based on various degrees of experience. They could feature either as additions to certifying packages or as dedicated workshops (Eventbrite, 2017).

Alongside equipment failure, running out of gas was either experienced or witnessed as top incident/accident. However, drowning, which can be a direct consequence of running out of gas, was not seen as representing a major threat. Campaigns promoting the rule of thirds and other standard safety procedures aimed at preventing incidents/accidents concerning running out of gas (and consequent drowning) are strongly recommended. These can be introduced by safety organisations, although the importance of breathing-gas management must be stressed during training. The scuba diving industry can benefit from the development of innovative devices that alert divers and prevent them from running out of gas. Safety agencies, in collaboration with engineering companies, are working on prototypes to address this need (Altepe et al., 2017). Devices can be designed to serve a variety of alerting purposes, for example those concerning buddy separation or being lost at sea, both of which were either experienced or perceived as top incidents/accidents in this study.

In line with recent research (Ranapurwala et al., 2017), buddy-system failure was either experienced or witnessed as a main incident/accident. This result contrasts with the importance given by scuba divers to the pre-dive buddy cheque. In addition, although scuba divers gave importance to the proper selection of a buddy, the buddy system and pre-dive buddy cheque, the buddy cheque controls were underestimated. These results call for action regarding the control of buddy-system functioning (Coleman, 2007; Ranapurwala et al., 2017). Pre-dive buddy cheque procedures should be created with the consensus of dive organisations to standardise buddy-system protocols and minimise risks during dives. The code of conduct of buddy pairs or diving groups needs to be enforced by certifying agencies and dive masters. Some notable agencies, for example Global Underwater Explorers and Unified Team Diving, give prime importance to pre-dive group cheques, but others may still require more emphasis to be placed on these procedures.

### Overarching Actions to Enhance Safety Culture in Recreational Scuba Diving

Both dive centres’ and scuba divers’ attitudes require action on various levels, involving dive centres all the way up to safety organisations and certifying agencies. Based on data and discussions retrieved from the available literature, it is evident that overarching programmes and actions are required to manage safety risks during scuba diving operations, raise awareness of safety among scuba divers, educate and equip scuba divers with important knowledge for managing risks, and enhance the sustainability of scuba diving activities (Wilks and Davis, 2000; Coxon et al., 2008).

This set of actions is embodied in programmes such as the Hazard Identification and Risk Assessment (HIRA) and Diving Safety Officer (DSO) (Burman, 2015, 2016a,b; Divers Alert Network [DAN] Southern Africa, 2016). These programmes, which were launched in 2015 by DAN Southern Africa, are applicable to all infrastructure, vehicles, vessels, equipment, and services pertaining to scuba diving activities, with particular (but not exclusive) focus on recreational scuba diving operations. HIRA is a campaign designed by DAN to equip scuba diving operators with knowledge to identify and mitigate risks associated with their business, and to implement emergency action/assistance plans (Burman, 2015, 2016a, 2017; Divers Alert Network [DAN] Southern Africa, 2016). The DSO campaign is a programme educating and empowering competent people to enhance and oversee the implementation of the HIRA by raising awareness, enhancing risk management and mitigating health and safety risks at the dive centre and among scuba divers (Burman, 2016b). The overarching aim of both campaigns is to create a culture of safety among dive centres and scuba divers, which is underpinned by commitment toward safety, concerns for risks and their potential impacts, consciousness of risks, and a sustained effort to reduce or manage these risks. A culture of safety leads to the natural improvement of safety on behalf of scuba diving operations and, ultimately, scuba divers who see that the dive centre is prioritising safety (Burman, 2016b).

### Study Limitations

There are a number of limitations associated with this study, which attention in order to properly interpret the results, and should be addressed in answering future research questions.

The study remained focused mainly on DAN members through convenience sampling, although the snowball sampling technique allowed the researchers to reach individuals beyond DAN members. The techniques of data collection online via convenience and snowball sampling hold the disadvantage of attracting a specific group of people, in this case, a potentially safety-aware group of scuba divers. In addition, many divers may have been guided to provide answers based on social norms, rather than based on their actual opinion and behaviour. This study used Likert scales in order to collect perception data from scuba divers. While Likert scales usefully provide information on the strength of a feeling or attitude, they also present some disadvantages (Maree and Pietersen, 2016). These include the provision of too simple answers, the provision of answers to questions even in cases where these have been misunderstood, and the limited number of options available in response to a given question, among others.

The psychological profile of scuba divers is an important component to measure in studies of this type; however, the extent to which psychological profile was assessed in this study only included safety attitudes and some self-reported behaviours (e.g., diving without a buddy). This study also focused on perceived risks, and its recommendations were based on these perceived risks. Perceived risk does not necessarily correlate with actual risk; therefore, the results in this study and the relevant interpretations must be considered with caution. An analysis of the influence of diver experience (e.g., technical divers vs. recreational divers) on perceptions was not within the scope of the paper. Indeed, the paper deals with universal recommendations based on scuba divers’ perceptions, regardless of the specialisation level of scuba divers. However, future research should consider the importance of analytical techniques (e.g., regression analysis) aimed at assessing the influence of scuba divers’ demographic and experience profile on their perception of safety and risk.

## Conclusion

This study investigated perceptions about safety in recreational scuba diving operations. Safety attitudes at dive centres and among scuba divers were assessed to recommend actions aligning perceptions by dive centres and scuba divers on safety, and to enhance risk prevention and management during diving operations. The results demonstrate that the middle-aged, experienced market dominating scuba diving activities gives prime importance to safety and the responsiveness of service providers, represented here by the dive centres. However, scuba divers underestimate the importance of a personal emergency action/assistance plan and of safety procedures, including the buddy system. Scuba divers agree that some risks, for example those associated with running out of gas, deserve attention. Dive centres’ perceptions of safety partly align with those of scuba divers. However, greater responsibility in raising awareness and educating scuba divers is required through participation in prevention campaigns and training. This study supports the introduction of overarching programmes such as the HIRA protocol for dive centres and scuba divers, and the DSO programme, intended to create awareness, improve risk management, and mitigate health and safety risks. These types of programmes have the goal of creating among dive centres and scuba divers a culture of safety, which is one aspect possessing the potential to support the sustainable future of recreational scuba diving.

## Ethics Statement

This study was conducted in accordance with the Declaration of Helsinki (World Medical Association, 2013) and was approved by the Academic Ethical Committee of Brussels (B200-2009-039). No private personal information was asked from participants in the study. The data were handled according to the Italian Law on privacy.

## Author Contributions

SL: contributed to the acquisition, analysis, or interpretation of data for the work (surveys), and wrote the submitted manuscript. SE and MP: contributed to the conception and design of the work (surveys), to the acquisition, analysis, or interpretation of data for the work (surveys), and reviewed the manuscript. FB: contributed to the conception and design of the work (HIRA and DSO), to the acquisition, analysis, or interpretation of data for the work (surveys), and reviewed the manuscript. TO: contributed to the analysis of data for the work (surveys). DC and GT: contributed to the conception and design of the work (surveys), and reviewed the manuscript. AM: oversaw the research programme, and reviewed and approved the manuscript. MS: reviewed the manuscript.

## Disclaimer

Divers Alert Network has no commercial interest in any of its safety programmes, especially not with the HIRA and DSO. These are intended as a service to the industry in the interest of meeting DAN’s mission of safety in diving. They are provided without cost.

## Conflict of Interest Statement

The authors declare that the research was conducted in the absence of any commercial or financial relationships that could be construed as a potential conflict of interest.

## Footnotes

1 www.greenbubbles.eu
